# Soil Moisture Content Estimation Based on Sentinel-1 and Auxiliary Earth Observation Products. A Hydrological Approach

**DOI:** 10.3390/s17061455

**Published:** 2017-06-21

**Authors:** Dimitrios D. Alexakis, Filippos-Dimitrios K. Mexis, Anthi-Eirini K. Vozinaki, Ioannis N. Daliakopoulos, Ioannis K. Tsanis

**Affiliations:** School of Environmental Engineering, Technical University of Crete, Chania 73100, Greece; philip.mexis@gmail.com (F.-D.K.M.); anthirini@hydromech.gr (A.-E.K.V.); daliakopoulos@hydromech.gr (I.N.D.); tsanis@hydromech.gr (I.K.T.)

**Keywords:** soil moisture content, Sentinel-1, Landsat 8, artificial neural network, HEC-HMS, Crete

## Abstract

A methodology for elaborating multi-temporal Sentinel-1 and Landsat 8 satellite images for estimating topsoil Soil Moisture Content (SMC) to support hydrological simulation studies is proposed. After pre-processing the remote sensing data, backscattering coefficient, Normalized Difference Vegetation Index (NDVI), thermal infrared temperature and incidence angle parameters are assessed for their potential to infer ground measurements of SMC, collected at the top 5 cm. A non-linear approach using Artificial Neural Networks (ANNs) is tested. The methodology is applied in Western Crete, Greece, where a SMC gauge network was deployed during 2015. The performance of the proposed algorithm is evaluated using leave-one-out cross validation and sensitivity analysis. ANNs prove to be the most efficient in SMC estimation yielding R^2^ values between 0.7 and 0.9. The proposed methodology is used to support a hydrological simulation with the HEC-HMS model, applied at the Keramianos basin which is ungauged for SMC. Results and model sensitivity highlight the contribution of combining Sentinel-1 SAR and Landsat 8 images for improving SMC estimates and supporting hydrological studies.

## 1. Introduction

The assessment of Soil Moisture Content (SMC) is indispensable for various disciplines such as meteorology, hydrology and agriculture [1], finding applications in evapotranspiration estimation, flood-risk prediction and assessment of irrigation requirements. The most accurate approach for SMC estimation is that of the gravimetric method [2], nevertheless, large scale SMC ground measurements are time and labor intensive. However, remote sensing provides a fast alternative to mapping SMC and its temporal distribution. The advent of satellite based remote sensing has led to a considerable amount of scientific literature on identifying the potential of such sensors to provide explicit SMC maps from space [3]. Several theoretical approaches exist for calculating backscattering from land surfaces with different roughness scales [4] and comparing them against in situ SMC observations.

Backscattering measurements of microwaves from SAR sensors have demonstrated their potential for effective monitoring of soil properties. In this context, research activities have shown that sensors operating within microwave bands from P to L are more sensitive to variations of the soil layer’s moisture level [5,6]. While at higher frequencies, such as C band or higher, radar’s ability to monitor soil moisture is mainly limited by vegetation cover, this problem is significantly reduced at lower frequencies [7]. Many factors, such as alterations in topography, vegetation regime, soil types, and water content, affect the spatial variability of SMC [8]. According to [9], soil properties and topography are the most significant physical parameters that jointly control the spatio-temporal evolution of soil moisture. Especially in the case of bare soils and high radar frequency observation, backscattering is considered to be extremely sensitive to soil surface roughness [10]. For reference, an observed surface is considered smooth if its elevation variations are smaller than the radar’s wavelength. While SMC is usually characterized by smooth changes in space and abrupt changes in time, soil roughness may drastically change spatially though it remains relatively constant in time [11]. In the case of high frequencies, backscattered signal is mostly affected by surface roughness and vegetation, rather than SMC. Various backscattering models have been developed over the past 30 years, categorized into three main groups: physical, empirical and semi-empirical [3]. Despite the progress achieved by refined electromagnetic models, the parameterization of surface roughness in soil backscatter modelling has not yet matured [12]. The accuracy of SMC estimation by inverse backscattering models is also affected by various parameters such as surface roughness [10,13].

Nowadays, most SAR systems operate at bands C and X (RADARSAT2, COSMO SkyMed, and TerraSAR-X), which are not considered optimal for retrieving SMC since they respond to surface roughness and vegetation cover as well [14]. Given the high temporal sampling and the operational configuration of the newly launched Sentinel-1 (C-band), this satellite is expected to make significant contributions in the operational monitoring of dynamic hydrological processes [15]. Compared to ENVISAT ASAR GM mode (stopped working in 2012), Sentinel-1 IWS mode is of improved spatial, temporal, and radiometric resolution while working at nearly the same center frequency (5.4 versus 5.5 GHz) [16]. Furthermore, according to [14], multi-temporal approaches for extracting SMC from C-band can successfully account for surface roughness effects and low vegetation cover. This limitation has been overcome with the Sentinel-1 mission, where sufficiently frequent SAR acquisitions are available to ensure the stability of roughness and vegetation regime among them.

Different approaches have been followed in the past to calculate SMC, such as multi-temporal change detection [17,18,19] and forward model inversion models (ANN, Bayes theorem) with extremely promising results. Concerning ANNs, their effectiveness in solving inverse remote sensing problems such as those required by SMC monitoring has diachronically been proved [20]. [21] have implemented the SMOSAR algorithm for retrieving SMC from Sentinel-1 multi-temporal data. A wide range of theoretical and empirical models for retrieving SMC from active and passive remote sensing data have been also utilized [22,23]. Among them, one of the most commonly adopted theoretical models is the Integral Equations Method (IEM) Model [24] and its evolution, the Advanced Integral Equation Model (AIEM) [25,26]. This model allows the simulation of the radar measurements in the presence of a specific soil condition, usually represented in terms of dielectric permittivity and roughness [27]. Concerning semi-empirical models, the most popular are those of Oh [28,29], providing analytical relationships between the backscattered radar signal and various physical soil parameters.

In water resources management, SMC is an essential variable playing a crucial role in most of the hydrological processes [30]. Hydrological models typically contain a substantial number of conceptual parameters that are difficult to measure directly. These parameters need to be calibrated to the best-fitting local values so that some agreement between the calculated and observed variables can be obtained. The idea of feeding hydrological models with input derived from satellite data may assist in overcoming such limitations and uncertainties of hydrological modeling [31]. [32] demonstrated that the Advanced Microwave Scanning Radiometer for EOS (AMSR-E) SMC product was essential for the calibration of the soil hydraulic properties in the Noah land surface model (LSM). [31,33,34] also showed that the calibration of a hydrological model with Scatterometer on European Remote Sensing Satellite (SCAT) improved soil moisture simulations at basin scale [35].

This study investigates the potential of the synergetic use of the newly-launched Sentinel-1 IW mode and Landsat-8 Operational Land Images (OLI) images for estimating the top 5 cm SMC. According to [11], the combined use of active and passive remote sensing data can provide complementary information in terms of soil water content. Vertical (VV) multi-temporal C-band ASAR backscatter measurements are used. Ground SMC measurements were collected during 2015 in a study area in Western Crete. The free, full, and open data policy of Sentinel-1 images adopted for the ESA Copernicus program opens new horizons to the extensive use of radar satellite remote sensing data. A non-linear Artificial Neural Network (ANN) approach is assessed for its potential to translate satellite remote sensing input to SMC. The estimated SMC values are used as input to the HEC-HMS [36] hydrological model to simulate a flow event that took place on April 2015 in the Keramianos sub-basin located in Chania, Crete, Greece. Model sensitivity to SMC spatial aggregation is also investigated.

## 2. Case Study and Data

**Figure 1:**
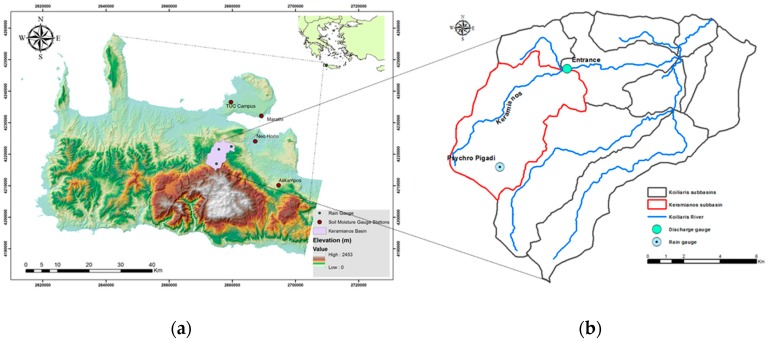
The broader study area, its topography and location of SMC and rain gauge stations (**a**). The Keramianos sub-basin used for the hydrological application, along with discharge and rain gauge locations (**b**).

The study site is located in the broader Chania region in the western part of the island of Crete, Greece. The climate of the study area is sub-humid Mediterranean with humid winters and warm summers [37]. The municipality of Chania covers a total area of approximately 2.343 km^2^. The Koiliaris watershed is located at the north of the municipality (Figure 1a), covers 130 km^2^ and has been characterized as a Mediterranean Critical Zone Observatory (CZO) due to its special geomorphologic and hydrogeological characteristics [38,39]. The Keramianos ephemeral tributary that drains the homonymous sub-catchment of 32 km^2^ includes a rain and stage gauge. Overgrazing in the Keramianos sub-catchment (Figure 1b) leaves the top soil vulnerable to surface runoff [40]. As a result of poor vegetation cover and steep slopes, the Keramianos tributary is often responsible for Koiliaris River flash flooding, which in the past has caused several damages in the downstream area of the Koiliaris watershed [41]. Here we have chosen to model a flow event of April 2015 in order to capture the importance of SMC even in low flow estimation.

### 2.1. Earth Observation Data

**Figure 2:**
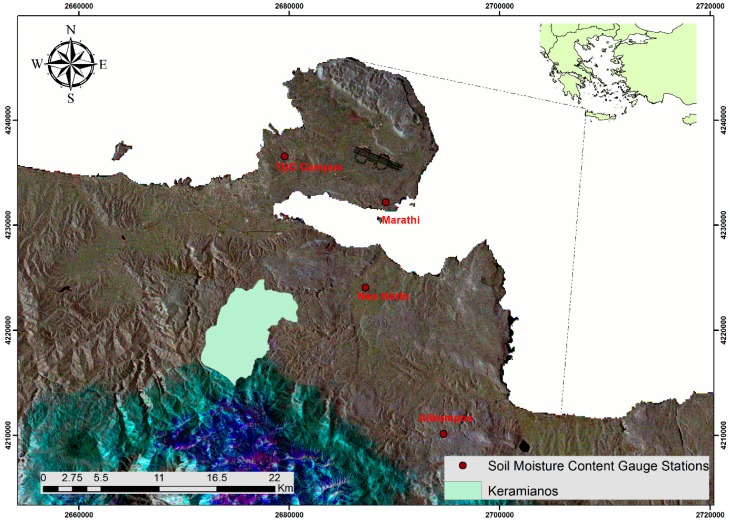
Sentinel-1 False Color Composite (RGB: 123; band 1: 21 February 2015, band 2: 12 April 2015, band 3: 21 July 2015).

For the needs of the study, 38 images of both radar (Sentinel-1) and optical/ thermal (Landsat 8) satellite sensors were collected (Table 1). Specifically, an integrated time series of satellite data covering the period from January 2015 to December 2015 was processed (Figure 2). Concerning Sentinel-1 images, the Interferometric Wave (IW) mode images were incorporated in the final model. In order to meet the demanding image quality and swath width requirements, the IW mode is used as a Scan SAR mode. This requires a fast antenna beam steering in elevation for Scan SAR operation [42]. Sentinel-1 incidence angles for western Crete mainly range between 38° and 41°. Although low incidence angles are considered to be optimal for SMC estimation, [43] argue that high incidence angles (>45°) are suitable for the discrimination between smooth and rough areas. Therefore, incidence angles were considered sufficient for this study. Furthermore, 11 cloud-free Landsat 8 images from the same period were acquired and analyzed to extract vegetation coverage and temperature regime at the four experimental fields.

Initially, geometric corrections are carried out using an adequate number of Ground Control Points (GCPs). The SRTM 3Sec is used to eliminate topography related phase changes. Due to topographical variations of a scene, distances are usually distorted in the SAR images. Range Doppler Terrain Correction is therefore applied to all SAR images to implement orthorectification. The SAR images are then first multi-looked to reduce speckle and radiometrically calibrated to derive the backscattering coefficients $\sigma^{0}\;\mathrm{in}\;\mathrm{dB}$. In terms of roughness and vegetation correction, the semi-empirical Dubois model [29] is implemented. The model is initially formulated using scatterometer data collected at six frequencies between 2.5 GHz and 11 GHz and has been used in many studies with generally satisfactory results [44]. The validity of the model is restricted to normalized surface roughness and incidence angles greater than 30°. The Dubois model has two equations [45] that relate the backscatter to sensor and soil surface parameters, one applicable to HH polarized data (horizontal transmit and horizontal receive) and the other for VV polarized data (vertical transmit and vertical receive). Concerning Sentinel-1, only VV polarized data are available. The VV equation is sufficiently assuming vegetation at early growth stages and low density. The parameter RMS height *s* is replaced by roughness mode. All the study areas are covered by weeds with mean height of 3 cm as measured in the field. For this purpose, a Vernier caliper was utilized. Significant variations were observed between different field campaigns mainly due to rain effect and cultivation practices.

The final model is modified to be used along with Topp’s model [46] (Equation (1)) which does not require any prior knowledge about soil other than ε to derive SMC, $\theta_{\nu }$:

(1) $$\theta_{\nu }=-\;5.3\times 10^{-2}+2.92\;\times \;10^{-2}\varepsilon \;-\;5.5\times 10^{-4}\varepsilon^{2}\;+\;4.3\;\times \;10^{-6}\varepsilon^{3}$$

Landsat 8 images are radiometrically corrected to account for illumination’s changes and changes in viewing geometry among different image acquisitions. Thus, the Digital Number (DN) values of satellite images are converted to reflectance values [37]. Eventually, images are corrected for atmospheric distortions using the Darkest Pixel (DP) atmospheric correction approach [47] and dams and lakes as non-variant Darkest Pixel targets. The method can practically eliminate atmospheric distortions of otherwise unknown distributions and intensities by accounting for dark and non-variant targets located in the image or by conducting in situ measurements.

The Normalized Difference Vegetation (NDVI) is used to estimate the degree of vegetation coverage in terms of vegetation height and density. Tall or dense vegetation absorbs most incident visible light and reflects a large portion of the near-infrared (NIR) light, resulting in high NDVI values [48]. NDVI is widely accepted as a sensitive indicator that can be used to monitor phenological variations and biomass changes of vegetation in time-series analyses [49].

The canopy temperature can indirectly describe the soil moisture regime [50]. The Thermal Infrared Sensor (TIRS) onboard the LandSat-8 mission measures land surface temperature in two thermal bands by applying quantum physics principles. Atmospheric conditions and their effects on Thermal Infrared (TIR) spectral band data are different from day to day, so images that are acquired on different dates often have different ranges in TIR values. Thus, a relevant index is developed to remove or reduce the absolute differences by normalizing the values to a range of 0 to 1 [51]. For this purpose, the thermal band (band 10) of Landsat 8 is used. The TIR_n_ was estimated for all the available Landsat 8 images and was incorporated as a parameter in the overall model.

### 2.2. Ground Data

**Figure 3:**
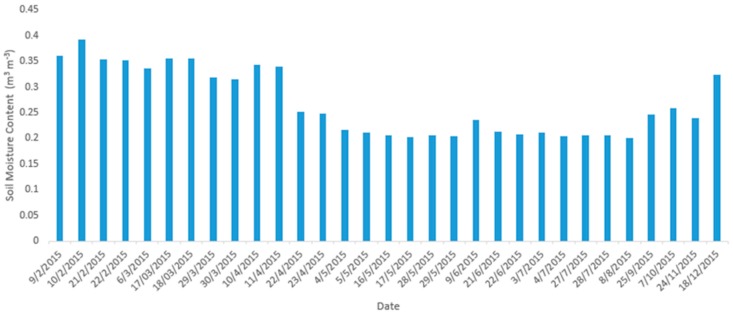
Temporal distribution of SMC measurements at Neo Horio gauge station.

Time Domain Reflectometry (TDR) Decagon EC-5 sensors were used to measure in situ SMC. EC-5 consists of two parallel-pronged plastic rods of 50 mm length and 9.8 mm width, and a spacing of 12.1 mm with a reported measurement volume of 0.24 L. The sensor reads mV and a compatible data logger converts mV readings into digital signal [52]. It operates at a frequency of 70 MHz with a measurement range between 0 and 0.57 m^3^·m^−^^3^ at a resolution of 0.1 m^3^·m^−3^. Sensors were installed in four experimental fields, serving as replicates, at the Eastern part of the Municipality of Chania: Marathi, Neo Horio, Alikampos, and at the campus of the Technical University of Crete (TUC) (Table 2, Figure 1a). All four experimental fields are areas of generally bare soil surface or very sparse vegetation (approximately 3 cm height). Sensors were installed approximately 5 cm below ground and measurements were collected every 15 min. Figure 3 shows daily averages for the period of the study from the Neo Horio TDR sensors. SMC measurements corresponding to the exact time of earth observation from Sentinel-1 were eventually extracted from the dataset. According to the overall number of available satellite images, 160 ground SMC measurements were incorporated in the overall analysis together with corresponding satellite data. In order to spatially reference SMC measurements against satellite images, their geographical locations were determined using a pair of differential GPS (DGPS) Leica GS20 Professional Data Mappers. DGPS measurements were corrected offline using the L1 pseudo-range in conjunction with station TUC2 from the Reference Frame Sub-Commission for Europe (EUREF) Permanent Network (EPN), located within the TUC campus. The same method was used to collect GCPs within the study area in order to geometrically correct the satellite images.

## 3. Methodology

**Figure 4:**
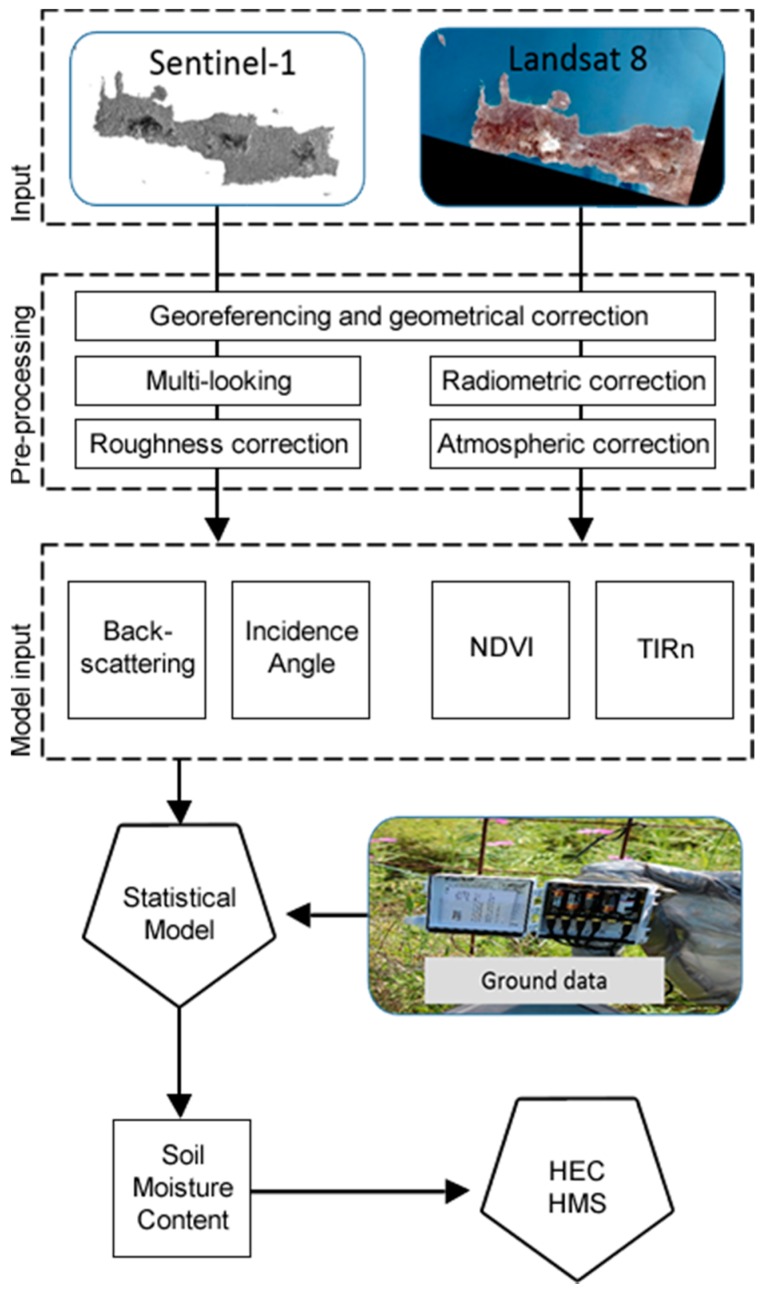
Flowchart of the overall methodology.

The overall methodology is based on the synergistic use of Sentinel-1 images and auxiliary Landsat 8 products to estimate SMC. Specifically, a SMC gauge network established in the Municipality of Chania in Western Crete is used as ground truth. Following, Sentinel-1 and Landsat 8 images are statistically analyzed using a non-linear Artificial Neural Network (ANN) approach. Four different parameters were selected as inputs in the ANN approach, namely, corrected radar backscattering $\sigma_{dB}^{0}$, NDVI, incidence angle ϑ, and thermal infrared temperature TIR_n_. Besides the first three variables that have been extensively used as input in previous studies, temperature has been incorporated in relevant models only either as Temperature-Vegetation-Drought-Index (TVDI) [53] or as a parameter that depends on dielectric properties and therefore on soil moisture [54]. Following, the estimated SMC is used as input in the HEC-HMS model to conduct hydrologic simulations. Figure 4 outlines the methodological steps presented in the following paragraphs.

### 3.1. ANN Approach

ANNs provide an alternative to conventional numerical modeling techniques, which sometimes are limited by rigid normality and linearity [55,56,57]. An ANN consists of a number of hidden neurons or nodes that work in parallel to convert data from an input to output layer. Here, the VV backscattering, NDVI, TIR_n_ and Incidence Angle parameters were used as input and ground SMC measurements (experimental data) as output in MATLAB^®^ environment. Besides backscattering data, NDVI was incorporated in the model for accounting vegetation and roughness, incidence angle for topography and thermal infrared temperature for water content. A feed-forward Multilayer Perceptron (MLP) modelis used. In MLPs, successive layers of neurons are interconnected, with connection weights that control the strength of the connection. The feasibility of ANNs in solving remote sensing problems has been highlighted in various studies since ANN can easily merge data coming from different sources into a unique integrated algorithm [58]. Specifically, the effectiveness of the ANN in estimating SMC has been investigated in other relevant studies [14,58] where the need for auxiliary information derived from passive remote sensing imagery such as the NDVI was pointed out. The optimal architecture of an ANN is defined by varying the number of neurons in the hidden layer and successively training and testing against variable sets previous unknown to the network. The main aim of the training process is to minimize the error between the ANN output and the input data by adjusting the correlation weights between them [59].

Trial and error (hidden layers and neurons added or removed from the model) determined the optimal MLP architecture to a three-layer network consisting of an input layer (four neurons: V backscattering, NDVI, TIR_n_, Incidence Angle), one hidden layer (10 neurons) and one output layer (in situ SMC). The specific architecture guaranteed an optimum model performance (minimum error and maximum convergence), avoiding any possible overfitting. The model was trained with the use of both experimental and simulated data in order to minimize the RMSE. Thus, 160 sets from each individual parameter (160 × 4 = 640 in total) were incorporated as input (experimental data) in the ANN model with 160 corresponding ground SMC values as output. The available data were divided in 80%-10%-10% for training, testing and validation phases, respectively. For the needs of training, 1000 iterations were set as a threshold to cease the procedure. Training was based on the Levenberg-Marquardt method which is an alternative of the Newton algorithm for finding an optimum solution to a minimization problem. The specific algorithm is often characterized as more stable and efficient [60]. ANN training was repeated 50 times and the RMSE of the mean value of the final results was estimated.

### 3.2. Hydrological Model

Continuous flow and precipitation time series data for the studied extreme event were available from the “Entrance” and “Psychro Pigadi” gauge stations, respectively (Figure 1b). The model was initially calibrated using past events from the period 2013–2014 and subsequently implemented for the flow event on April 2015. The total observed rainfall recorded in the gauge station “Psychro Pigadi” for the studied period was 67.8 mm. The maximum observed flow was 2.67 m^3^ s^−1^ and the observed water volume equivalent for the sub-catchment was 1.197 mm. Furthermore, SMC was converted to saturation degree S using soil porosity value ε=0.5094, for the specific sub-catchment as obtained from [61].

For the need of the study the Hydrologic Modeling System (HEC-HMS) was used. HEC-HMS has been developed by the Hydrologic Engineering Center (HEC) of the US Army Corps of Engineers to simulate the hydrologic processes of dendritic watershed systems [36]. Since moisture conditions are a crucial factor for flood modeling in the Mediterranean region [62] but uncertainties are high, the loss method adopted here is the simple but robust Soil Moisture Account (SMA) [36]. SMA uses one vegetation (canopy interception storage) and three ground (soil, upper groundwater, and lower groundwater storage) reservoirs to represent the vertical dynamics of soil moisture. Here, emphasis is given to the soil profile storage, which represents water stored in the top layer of the soil. Its principal input parameter is the initial condition of the soil, which is determined as the degree of saturation S (%) at the beginning of the simulation. Here, we consider that S can be estimated directly from the SMC derived from the satellite data and the soil porosity ε (dimensionless) using:

(2) $$S=\frac{\theta_{\nu }}{\varepsilon }\;\times \;100$$

When infiltration capacity is reached, excess precipitation is generated. The Snyder (1938) synthetic unit hydrograph (UH) method which relates the watershed’s physical characteristics to the basic parameters of the UH is used for transforming excess precipitation to runoff. Subsurface calculations are performed using the recession base flow method, which is intended for event simulation. Finally, the Muskingum routing method performs the calculations in the reach using a simple conservation of mass approach to route flow through the stream reach, whereas interactions with the subsurface are performed by the constant loss/gain method contained within the reach.

Three criteria are mainly used to assess the effectiveness of each network and its ability to make precise predictions: RMSE indicates the discrepancy between the observed and calculated values, and R^2^ representing the percentage of the initial uncertainty explained by the model. The performance of the hydrological model is assessed using Nash-Sutcliffe efficient (NSE) coefficient [63] which is a measure of the model quality with respect to the representation of the variance of the data:

(3) $$NSE=1\;-\;\frac{\sum^{\text{​}}{\left(y_{i}-\hat{y_{i}}\right)}^{2}}{\sum_{i=1}^{n}{\left(y_{i}-\bar{y}\right)}^{2}}$$

where $\bar{y}$ is the mean value of the n observations. The perfect fit between observed and calculated values would have RMSE = 0, R^2^ = 1 and NSE = 1. Finally, absolute percentage error (APE) given by $APE\;=\;\left|\left(y_{i}-\hat{y_{i}}\right)/y_{i}\right|$ is also used to assess hydrological model performance.

## 4. Results and Discussion

**Figure 5:**
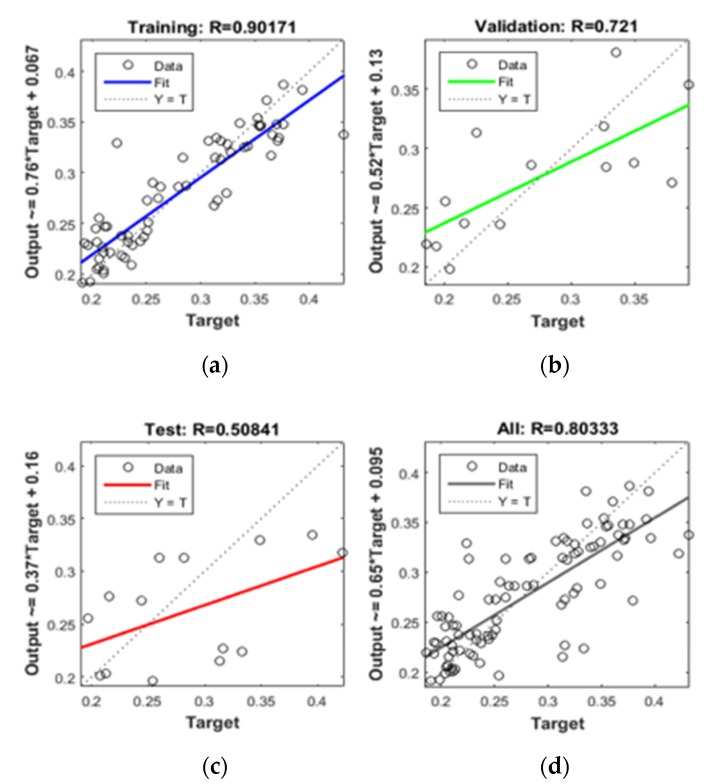
Example of ANN statistics (R in terms of (**a**) training, (**b**) validation, (**c**) testing and (**d**) overall performance).

The ANN MLP simulation results (Figure 5) produced good agreement with the ground truth data in terms of R^2^ and RMSE (Table 3).

In order to assess the model’s performance, the leave-one-out cross validation method is applied. Specifically, each time the ground SMC measurements of a SMC gauge station is treated as prediction points and compared to the output of the ANN model. Results show that the elimination of the TUC field from the overall model improved its performance. Most importantly, the fact that a single network can yield acceptable results for all fields underlines the ability of ANNs to spatially extrapolate SMC estimates. In addition, the comparison of observed and calculated SMC measurements highlighted the fact that the optimal performance of the ANN network is observed during summer and spring rather than winter and autumn period. Finally, a sensitivity analysis is performed to define the relative importance of each individual parameter in the SMC detection methodology (Table 4). Results denote that among the different input parameters, NDVI is the most important for the smooth performance of the ANN. However, the final results highlight the need of the synergistic use of the four individual parameters for the optimum performance of the model.

**Figure 6:**
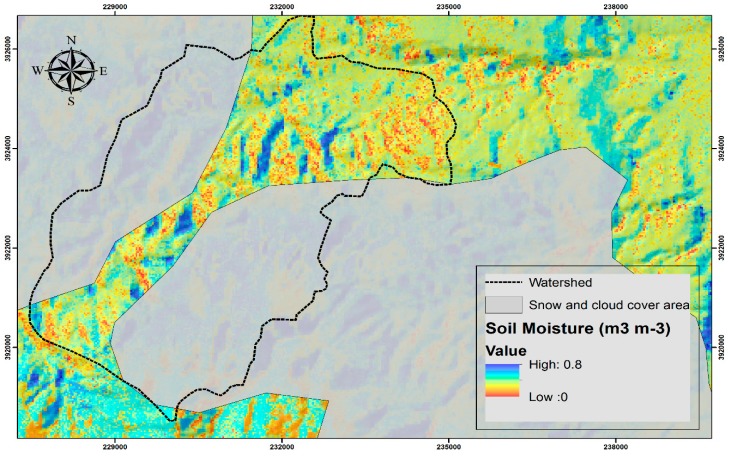
Soil Moisture Content map in Keramianos basin and broader area. The snow and cloud cover area is indicated with grey colour.

The flow event on April 2015 was investigated in order to apply the saturation degree values obtained from the satellite SMC measured on 10 April 2015. For this purpose, the already trained ANN model was implemented over Keramianos watershed (ungauged in terms of SMC) grid cells using the same parametrization and producing a spatially distributed SMC estimation. The final developed map (30 m pixel resolution) was spatially masked due to the partial cloud and snow cover of Landsat 8 image within Keramianos watershed during flow event (Figure 6).

Since the SMA method implementation of HEC-HMS accepts a single value to describe the SMC of the lamped watershed, the average value of the estimated SMC grid cells (masked area) is used. Average SMC before the rain event is estimated at 0.3334 m^3^ m^−3^ (S = 65.5%) and for this value the simulation results yield a satisfactory performance when compared against observations (NSE = 0.712, R^2^ = 0.726; Table 5). For the average S the equivalent water volume is simulated at 1.05 mm, underestimating the observed volume by 12.5% (Table 5). In Table 5, model efficiency criteria are also shown for ±10% and ±20% variations to the average SMC value used as input in HEC-HMS. The simulated equivalent water volume is strongly affected by the satellite-derived SMC values, ranging from 0.57 mm (10% decrease of SMC average value) to 1.54 mm (10% increase of SMC average value), or −53% and +28% from the observed value, respectively.

**Figure 7:**
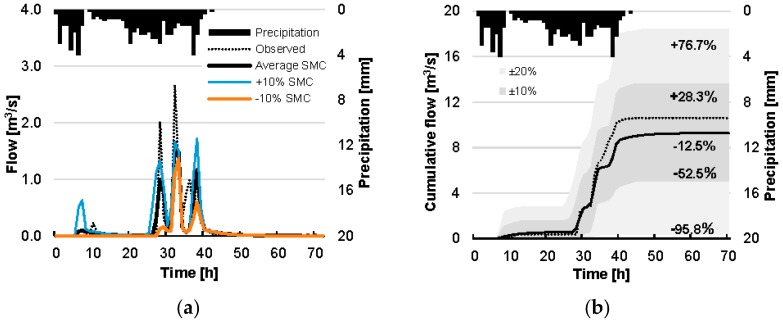
Instant (**a**) and cumulative (**b**) flow results from the HEC-HMS simulation. Right panel includes modelled estimates for ±10% and ±20% difference in SMC and the respective present difference in final cumulative flow. For clarity, left panel only includes estimates for ±10% difference in SMC.

Based on the results of Table 5, Figure 7a shows the modelled instantaneous flow for the average SMC value and the ±10% variation as a function of the observed flow time series (±20% not shown for simplicity). Figure 7b translates these values (including the ±20% variation) for cumulative flow values. The percentages in the Figure 7b demonstrate the increase or decrease of the respective simulated equivalent water volume versus the observed equivalent water volume value, also shown in Table 5. The simulated flow time series, using the initial average satellite SMC estimate, fits adequately with the flow observations (R^2^ = 0.726), whereas the percentage variation of the specific value denotes that model results are very sensitive to variations of the saturation degree. Besides this sensitivity, satellite-based SMC estimates are adequate to result into a good fit.

**Figure 8:**
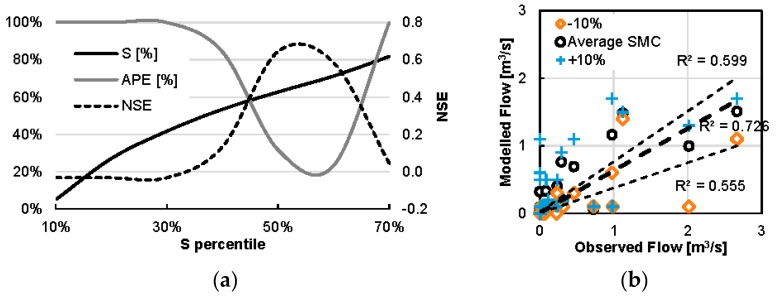
Sensitivity analysis of NSE and APE for the quantiles of the degree of saturation S as estimated from the spatially distributed satellite-derived SMC (**a**). Modelled versus observed values including modelled estimates for ±10% difference in SMC (**b**).

A more thorough sensitivity analysis is conducted by applying the entire range of the spatial variability of satellite-predicted SMC in the watershed to HEC-HMS. The behavior of the estimated NSE and APE of the resulting flow depending on the percentile of SMC used are shown in Figure 8. Values of SMC between percentiles 50–60% indeed yield acceptable values of model error. Beyond the range of percentiles 50–60% the model results deteriorate fast, which effectively shows the model sensitivity to the parameter of the degree of soil saturation. Moreover, the model generates no flow for S under 40% (which coincides with the 30% percentile of values in the watershed) thus providing a feel for the ability of the watershed to absorb runoff. This sensitivity of the hydrological model to small fluctuations of SMC underlines the importance in the contribution of high quality remote sensing data in reliable model output.

## 5. Conclusions

This paper investigates the estimation of surface Soil Moisture Content (SMC) using multisource and multi-temporal remote sensing data (Sentinel-1, Landsat 8) for use in hydrological applications. Specifically, it aims to highlight the potential of the newly launched Sentinel 1 sensor in estimating SMC. In addition, the proposed model incorporates four different input parameters in order to feed efficiently an ANN model for estimating SMC. Finally, the manuscript aims to highlight the potential of satellite remote sensing in providing essential input data to hydrological modeling. SMC is an indispensable input data for hydrology. Due to the fact that the collection of in situ SMC data in remote areas is often impractical or impossible, the development of alternative data collection methods is necessary.

The correction of SAR images in terms of vegetation effects and roughness was carried out by coupling Dubois and Topp model. Lack of HH polarization information from the Sentinel-1 possibly renders it applicable only for areas of low vegetation growth and density, which is nevertheless the very case for many flood prone watersheds. Statistical analysis of remote sensing variables versus ground SMC measurements showed that a non-linear approach can explain as much as 89.5% (Table 4) of data uncertainty for the specific case study. ANNs are certainly a good option for the problem at hand, being essentially non-parametric and requiring little understanding of the inner workings between model variables input and output.

Concerning environmental parameters, the results in our case study denoted the fact that incidence Angle ϑ is the least sensitive parameter, while NDVI is the most sensitive for the accurate estimation of SMC. In addition, the thermal infrared temperature parameter seems to be important for the overall performance of the model. Results demonstrate that the retrieval of SMC is possible at C-band (VV polarization) by using an inversion algorithm that includes a compensation for the effects of vegetation. Furthermore, it is possible that even the little vegetation present in the experimental fields is enough to induce attenuation in the measured backscattering. Hence, the complementary information from optical/thermal infrared sensors was proved substantial for the optimum performance of the model. Therefore, NDVI may be the most important parameter but all parameters are important for the optimum results.

Soil moisture is a crucial factor when evaluating the initial conditions for flood prediction, through an event-based rainfall–runoff model. The estimated SMC values for sub-basin ungauged for soil moisture were used for the hydrologic simulation of a flow event. In this study, the hydrological simulation parameterization has been improved through the use of satellite data permitting accurate prediction of flood characteristics. Simulations have shown that a calibrated model can be very sensitive to SMC, producing over 50% uncertain results even for low flows. Therefore, a precise estimation is indispensable in order to yield meaningful results. In conclusion, the sensitivity of the hydrological model results regarding the SMC values changes is evident, showing the importance of an accurate initial soil moisture condition determination through the use of multisource and multi-temporal remote sensing data (Sentinel-1, Landsat 8).

The presented methodology forms a sufficient method for the determination of initial soil moisture conditions during the hydrologic analysis. It can be also used as an alternative to the data intensive physical models. Furthermore, it highlights the potential of the ANN inversion model for the estimation of Soil Moisture Content from SAR observations. Future research will focus on further data collection for a more accurate validation of the results at a wider range of stream flows and vegetation cover.

## Figures and Tables

**Table 1:** Satellite data used in the study.

#	Sensor	Date of Acquisition	#	Sensor	Date of Acquisition
**1**	Landsat 8	9 February 2015	**20**	Sentinel-1	23 April 2015
**2**	Landsat 8	13 April 2015	**21**	Sentinel-1	4 March 2015
**3**	Landsat 8	29 April 2015	**22**	Sentinel-1	5 March 2015
**4**	Landsat 8	15 March 2015	**23**	Sentinel-1	16 March 2015
**5**	Landsat 8	31 March 2015	**24**	Sentinel-1	17 March 2015
**6**	Landsat 8	16 June 2015	**25**	Sentinel-1	28 March 2015
**7**	Landsat 8	18 July 2015	**26**	Sentinel-1	29 March 2015
**8**	Landsat 8	19 August 2015	**27**	Sentinel-1	9 June 2015
**9**	Landsat 8	20 September 2015	**28**	Sentinel-1	21 June 2015
**10**	Landsat 8	23 November 2015	**29**	Sentinel-1	22 June 2015
**11**	Landsat 8	25 December 2015	**30**	Sentinel-1	3 July 2015
**12**	Sentinel-1	16 January 2015	**31**	Sentinel-1	4 July 2015
**13**	Sentinel-1	17 January 2015	**32**	Sentinel-1	27 July 2015
**14**	Sentinel-1	9 February 2015	**33**	Sentinel-1	28 July 2015
**15**	Sentinel-1	29 March 2015	**34**	Sentinel-1	8 August 2015
**16**	Sentinel-1	30 March 2015	**35**	Sentinel-1	25 September 2015
**17**	Sentinel-1	10 April 2015	**36**	Sentinel-1	7 October 2015
**18**	Sentinel-1	11 April 2015	**37**	Sentinel-1	24 November 2015
**19**	Sentinel-1	22 April 2015	**38**	Sentinel-1	18 December 2015

**Table 2:** Spatial characteristics of Soil Moisture Content gauge stations.

Experimental Field	Distance from Sea (m)	Elevation (m)
**Marathi**	450	52
**Neo Horio**	3000	36
**Alikampos**	6000	398
**TUC Campus**	1500	120

**Table 3:** Statistics of R^2^ and RMSE concerning the performance of MPL ANN algorithm in the different SMC gauge stations.

#	Study Area	R^2^	RMSE
**1**	Marathi	0.867	0.022
**2**	Neo Horio	0.842	0.041
**3**	Alikampos	0.914	0.031
**4**	TUC	0.810	0.047
**5**	Overall (All the study sites)	0.500	0.042
**6**	Study Areas: Neo Horio, Marathi, Alikampos	0.829	0.040
**7**	Study Areas: Neo Horio, TUC, Alikampos	0.819	0.048
**8**	Study Areas: Alikampos, Marathi, TUC	0.657	0.033
**9**	Study Areas: TUC, Marathi, Alikampos	0.400	0.058

**Table 4:** Sensitivity analysis results of the input factors. Bold values inside brackets indicate the percent difference between the leave-one-out results and the results including all the parameters (according to Table 3).

Subtracted Parameter	Marathi		Neo Horio		Alikampos		TUC		All Fields	
	R^2^	RMSE	R^2^	RMSE	R^2^	RMSE	R^2^	RMSE	R^2^	RMSE
$\sigma_{db}^{0}$	0.724	0.057	0.846	0.055	0.867	0.036	0.811	0.044	0.745	0.044
	**(−17%)**		**(+0.4)**		**(−6%)**				**(+50%)**	
**NDVI**	0.338	0.052	0.569	0.059	0.506	0.072	0.552	0.082	0.349	0.069
	**(−62%)**		**(−35%)**		**(−35%)**		**(−32%)**		**(31%)**	
**TIR_n_**	0.774	0.039	0.895	0.028	0.664	0.066	0.781	0.053	0.509	0.062
	**(−10%)**		**(+6%)**		**(−28%)**		**(−4%)**		**(+1.8%)**	
ϑ	0.788	0.034	0.87	0.032	0.857	0.035	0.843	0.041	0.746	0.051
	**(−9%)**		**(+3%)**		**(−7%)**		**(+4%)**		**(+50%)**	

**Table 5:** Model results according to the degree of saturation *S* parameter change.

SMC Scenario	Satellite SMC (m^3^ m^−3^)	Degree of Saturation, S (%)	NSE	R^2^	Simulated Equivalent Volume, Q (mm)	APE (%)
−20%	0.2667	52.4	0.016	0.356	0.05	95.8
−10%	0.3001	58.9	0.494	0.555	0.57	52.5
Average SMC	0.3334	65.5	0.712	0.726	1.05	12.5
+10%	0.3667	72.0	0.556	0.599	1.54	28.3
+20%	0.4001	78.5	0.253	0.525	2.12	76.7
